# Aspartic and Glutamic Acid Templated Peptides Conjugation on Plasma Modified Nanofibers for Osteogenic Differentiation of Human Mesenchymal Stem Cells: A Comparative Study

**DOI:** 10.1038/s41598-018-36109-5

**Published:** 2018-12-04

**Authors:** Günnur Onak, Mustafa Şen, Nesrin Horzum, Utku Kürşat Ercan, Ziyşan Buse Yaralı, Bora Garipcan, Ozan Karaman

**Affiliations:** 1 Department of Biomedical Engineering, İzmir Katip Çelebi University, İzmir, 35620 Turkey; 2 Department of Engineering Sciences, İzmir Katip Çelebi University, İzmir, 35620 Turkey; 3 Institute of Biomedical Engineering, Bogazici University, 34684 İstanbul, Turkey; 4 Bonegraft Biomaterials Co., Ege University Technopolis, 35100 Bornova, İzmir Turkey

## Abstract

Optimization of nanofiber (NF) surface properties is critical to achieve an adequate cellular response. Here, the impact of conjugation of biomimetic aspartic acid (ASP) and glutamic acid (GLU) templated peptides with poly(lactic-co-glycolic acid) (PLGA) electrospun NF on osteogenic differentiation of human bone marrow-derived mesenchymal stem cells (hMSCs) was evaluated. Cold atmospheric plasma (CAP) was used to functionalize the NF surface and thus to mediate the conjugation. The influence of the CAP treatment following with peptide conjugation to the NF surface was assessed using water contact angle measurements, Fourier-Transform Infrared Spectroscopy (FTIR) and X-ray Photoelectron Spectroscopy (XPS). The effect of CAP treatment on morphology of NF was also checked using Scanning Electron Microscopy (SEM). Both the hydrophilicity of NF and the number of the carboxyl (-COOH) groups on the surface increased with respect to CAP treatment. Results demonstrated that CAP treatment significantly enhanced peptide conjugation on the surface of NF. Osteogenic differentiation results indicated that conjugating of biomimetic ASP templated peptides sharply increased alkaline phosphatase (ALP) activity, calcium content, and expression of key osteogenic markers of collagen type I (Col-I), osteocalcin (OC), and osteopontin (OP) compared to GLU conjugated (GLU-pNF) and CAP treated NF (pNF). It was further depicted that ASP sequences are the major fragments that influence the mineralization and osteogenic differentiation in non-collagenous proteins of bone extracellular matrix.

## Introduction

One of the primary aims of the bone tissue engineering is to fabricate scaffolds that are capable of providing adequate microenvironment similar to native bone tissue^1,2^. A variety of natural and synthetic materials have been used for constructing scaffolds in tissue engineering applications. Basically, the most frequently used natural materials for nanofiber (NF) fabrication can be classified as proteins and polysaccharides. The most commonly used proteins are collagen, fibronectin, fibrin and decellularized extracellular cell matrix (ECM). As for the polysaccharides, hyaluronic acid (HA) and chitosan (CS)/chitin are the most widely used natural scaffold materials. Although natural polymeric NF provide sufficient biological cues for bone regeneration, risks of immune system triggering, disease transmission and fast degradation along with poor mechanical properties limit the usage of these NF. Therefore, synthetic polymeric NF with adaptive biological, degradation and mechanical properties have attracted considerable attention for bone tissue engineering applications^3^. The most attractive synthetic polymers used for scaffolds in tissue engineering applications are Poly (L-lactic acid) (PLA or PLLA), Poly(lactide-co-glycolide) (PLGA) and Polycaprolactone (PCL). These synthetic materials have good mechanical and biocompability properties and are approved by Food and Drug Administration (FDA) for clinical use. A variety of criteria including strength, hardness, porosity, osteoconductivity and fabrication capability should be taken into consideration when designing a scaffold in bone tissue engineering^4^.

Electrospinning has been widely used to produce NF from natural and synthetic polymers to fabricate scaffolds. Electrospun NF properties can be easily tuned to mimic the natural structure of a bone with desired physical properties such as high porous and a large surface area which might subsequently enhance cellular behaviours such as cell adhesion, proliferation, and differentiation^5–8^. High surface to volume ratio is a typical characteristic of NF and plays an important role in providing bone-tissue mimetic morphology to enhance both cell-scaffold interaction and bone regeneration.

The absence of biological cues on synthetic NF arises the need of further surface modification. Most of the synthetic polymeric NF including PLA, PLGA and PCL do not have necessary surface chemistry to conjugate functional proteins or peptides due to high hydrophobicity^9^. Improved hydrophilicity along with the presence of particular functional groups on the surface of electrospun NF plays an important role in cell adhesion, proliferation and migration^10,11^. Various techniques such as pulsed laser deposition, ion beam deposition, covalent immobilization, photochemical modification, and plasma treatment have been used to modify the surface chemistry to improve hydrophilicity and introduce functional groups that can serve as biological cues^12–16^. Plasma is defined as fourth matter of state and composed of free electrons/radicals, electrically excited species, reactive oxygen/nitrogen species and UV photons^17^. Plasma formation occurs via ionization of a gas and could be artificially generated by the passage of electricity through the gas. When an external electrical field created under high voltage is applied to the gas, free electrons are first accelerated under the influence of applied electrical field. Accelerated electrons collide with gas atoms and/or molecules leading to removal of electrons from the structure of gas molecules and causing electron avalanche and ionization of the gas. When free electrons are first accelerated under the externally applied electric field, they gain kinetic energy and their temperature might rise up to thousands of kelvins. Kinetic energy of free electrons is transferred to gas molecules during collisions between free electrons and gas molecules. Depending on the efficacy of the energy transfer from electrons to heavier ions and the neutral gas atoms and/or molecules, plasmas are classified as thermal (or hot or equilibrium) and non-thermal plasma (or cold or non-equilibrium). In cold plasmas, cooling of heavier ions and neutral gas atoms and/or molecules is more efficient than the energy transfer from electrons to them^18,19^. Thus, the plasma remains in the room temperature and therefore, cold plasma could be applied to heat sensitive biological substrates and biomaterials. Reactive oxygen and nitrogen species (RONS) along with free radicals generated during formation of cold atmospheric plasma (CAP) could react with treated materials to obtain surface modification with no or minimal damage to surface^20^. Plasma-mediated surface modification is shown to improve cell attachment due to increase of hydrophilicity^21^. CAP has antimicrobial/anticancer activity and is an emerging technique with great potential to be used in various tissue engineering applications^22–24^.

Modification of a scaffold using bioactive molecules such as ECM proteins or peptides can activate desired signalling pathways and thus enhance functionality of scaffolds. Using peptides rather than native proteins have some advantages such as high resistance to pH or temperature changes, easier manipulation during grafting, low risk of pathogenic contamination/immune system triggering and chemical synthesis that provides precise control over the chemical composition of the peptide^25^. Peptides are used to modify the surface of the scaffold to provide biological ligands that can enhance cell adhesion, proliferation, differentiation and thus cell-scaffold interaction. Materials that are modified with peptides have been shown to be more effective in bone formation. Recently, several groups have used bone ECM proteins mimetic peptides in surface modification of NF and demonstrated the positive impact of such modification on both cell adhesion and osteogenic differentiation^26,27^.

Initiation of the mineralization process in bone regeneration is regulated by osteocalcin, osteopontin and bone sialoprotein^28^. These non-collagenous proteins contain largely aspartic acid (ASP) and glutamic acid (GLU) residues that are known to act as a nucleation point for calcium phosphate (CaP) mineralization^28^. There have been studies showing the individual effect of different repeating units of ASP and GLU sequences on mineralization and osteogenic differentiation^27,29–31^. For instance, Barati *et al*. investigated the influence of supplementing the nucleation buffer with various organic acids on osteogenic differentiation of hMSCs seeded on glutamic acid functionalized NFs. CaP nucleation depended on the acidic strength and the number of hydroxyl groups capable of hydrogen bonding on organic acids. According to the results, citric acid showed the highest CaP nucleation and osteogenic differentiation^32^. Ceylan *et al*. functionalized the surface of NF with Glu-Glu-Glu (EEE) and demonstrated that this short peptide promoted a more mature osteogenic differentiation than Asp-Gly-Glu-Ala (DGEA), an osteoinductive collagen I derived peptide known to improve initial adhesion, spreading and osteogenic differentiation for hMSCs^33^. Oslzta *et al*. reported that poly-Asp sequences act as an analogue of non-collagenous acidic proteins when deposited on purified collagen NF which resulted in significantly improved mineralization^31^. However, to the best of our knowledge, a comparative study that evaluates the effect of repeating sequences of ASP and GLU on osteogenic differentiation of hMSCs when used to modify the surface of synthetic NF has not been reported.

In this study, first the performance of using CAP for conjugation of different non-collagenous ECM proteins mimetic peptides with PLGA electrospun NF was investigated and then, the influence of ASP and GLU repeating short peptide sequences on bone regeneration was evaluated with respect to cytochemical, mRNA expression and immunofluorescence analysis. Electrospinning was used to fabricate FDA approved PLGA NF^34,35^. To promote the peptide-NF conjugation, CAP was adopted to introduce functional carboxyl (-COOH) groups on NF. The surface chemistry of the CAP treated and untreated NF was assessed using different techniques including Scanning Electron Microscopy (SEM), water contact angle measurements, and X-ray Photoelectron Spectroscopy (XPS). Since the composition of bone tissue ECM such as non-collageneous proteins plays a major role in osteogenic differentiation and mineralization, modification of NF surface with biomimetic peptides resembling the functional units of these proteins may accelerate mineralization and osteogenic differentiation.

## Materials and Method

### Peptide Synthesis

All chemicals used for peptide synthesis were purchased from AAPPTEC (Louisville, KY, USA). EEEEEE (Glu-Glu-Glu-Glu-Glu-Glu), DDDDDD (Asp-Asp-Asp-Asp-Asp-Asp), EEEEEEK (K:Lys), and DDDDDDK peptide sequences, hereafter denoted by GLU, ASP, GLUK, and ASPK respectively, were synthesized manually on 4-Methylbenzhydrylamine (MBHA) resin (0.67 mmol/g loading capacity) by using 9-fluorenylmethoxycarbonyl (Fmoc) chemistry as previously described^21,27^ (see chemical structures of peptides in Fig. S1, Supporting Information). All amino acid couplings were performed with 2 equivalents (based on loading capacity of resin) of Fmoc-protected amino acid, O-Benzotriazole-*N*,*N*,*N*′,*N*′-tetramethyluronium-hexafluoro-phosphate (HBTU; 2 equiv), hydroxybenzotriazole (HOBt; 2 equiv) and *N*, *N*-diisopropylethylamine (4 equiv; DIEA) HBTU in *N*,*N*-Dimethylformamide (DMF) for 3 hours. Fmoc-protecting group was removed using 20% piperidine in DMF for 30 minutes. Each coupling and deprotection reaction was monitored by ninhydrin test. For the conjugation of Fluorescein isothiocyanate (FITC) to the peptide, Mtt protecting group of –Lys (EEEEEEK) was removed by using solution containing trifluoroacetic acid (TFA) (0,047% (v/v)), triisopropylsilane (TIPS) (0.00125% (v/v)), deionized water (0.00125% (v/v)), and Dichloromethane (DCM) (0.95% (v/v)). The resin was washed 6 times with 10 mL of the solution for 5 minutes followed by the DCM wash. Finally, the resins were washed with 5% (v/v) DIEA in DCM. The FITC coupling solution containing 389.40 mg (FITC; Sigma Aldrich, St. Louis, MO, USA) and 256.8 μL DIEA was prepared in 3.0 mL DMF and added on the reaction vessel covered with aluminium foil in order to prevent the photobleaching of FITC. The peptide was cleaved from resin by using cleavage solution composed of TFA: TIPS: H_2_O solution at ratio of 95:2.5:2.5. TFA was evaporated on the rotary evaporator. The peptide was washed with ice-cold diethyl ether three times and freeze-dried. Next, purification of peptides was conducted via preparative high performance liquid chromatography (HPLC, Agilent 1200 series) system equipped with Zorbax Extend-C18 2.1 × 50 mm column. The mobile phase was used with the gradient of 0.1% TFA/water and 0.1% TFA/acetonitrile and detection wavelength was selected as 220 nm^21^. Mass spectrums of GLU, ASP, GLUK, and ASPK peptides were characterized via mass spectrometry (Agilent 6530 Q-TOF) with an electrospray ionization (ESI) source. (see liquid chromatography and mass spectra for GLU, ASP, GLUK, and ASPK in Figs S2 and 3 and Table S1, Supporting Information).

### Fabrication and Characterization of Nanofibers

A 7.0 wt% PLGA was prepared from PLGA (lactide/glycolide 50:50) powder and 1,1,1,3,3,3-hexafluoro-2-propanol (HFIP; Matrix Scientific; Columbia) solvent with stirring. The polymer solution was injected with a 5-mL syringe through a 21-gauge needle. The syringe was placed to syringe pump and the needle was connected to the positively charged electrode. The aligned NF were collected by 12 mm circular glass coverslips (VWR, Bristol, CT, USA) attached on an aluminium rotating wheel, powered by a high-speed direct current (DC) motor. The electrospinning was conducted using the following parameters; an electrical potential of 20 kV, 1.0 mL/h injection rate, 15 cm of needle-to-collector distance, and a rotation speed of 1200 rpm. Further details of the optimized processing parameters can be found elsewhere^27^. The NF were imaged with a Scanning Electron Microscope (SEM; Carl Zeiss Microscopy, Germany) at 3 kV accelerating voltage after coating with gold (QUORUM; Q150 RES; East Sussex; United Kingdom) at 20 mA for 60 seconds. The scale bars in the images were obtained from the SEM software and the fiber diameters and distributions were analysed with ImageJ software (National Institutes of Health, Bethesda, MD, USA) to determine the average fiber size.

### Peptide Conjugation to Nanofibers

CAP was applied on electrospun NF at 1.5 kHz frequency and 31 kV of output voltage for 45 seconds with a fixed 1 mm discharge gap. CAP treated and non-treated NF, hereafter denoted by pNF and NF, respectively, were thoroughly washed. 2-Morpholinoethanesulfonic acid buffer (MES; 0.1 M, pH 6.5) was prepared by adjusting the pH with 0.1 NaOH. EDC/NHS solution (2 mM EDC and 5 mM NHS) was prepared with 0.1 M MES buffer. NF were incubated in EDC/NHS solution for 40 minutes to create carboxyl-rich surfaces. Then, the NF was incubated in 1 mM ASP and GLU peptide in sterile Phosphate Buffer Solution (PBS) at 4 °C for 24 hours. The NF was thoroughly rinsed with PBS before characterization.

The effect of CAP treatment for 15 s, 30 s, 45 s, 60 s, 90 s and peptide (ASP and GLU) conjugation on pNF and NF surface on hydrophilicity was evaluated by water contact angle measurements using KSV Attension Theta goniometer (Biolin Scientific, Stockholm, Sweden). Briefly, 10 μL deionized water was dropped onto the nanofiber surface, photographed and the contact angle (θ) was calculated.

FTIR spectra of non-CAP treated NF, 45 s CAP treated NF, non-CAP treated peptide conjugated NF (GLU-NF, ASP-NF), and 45 s CAP treated peptide conjugated NF (GLU-pNF, ASP-pNF) were obtained using a Nicolet iS5 spectrometer (Thermo Scientific, Madison, WI, USA) with a spectral resolution of 4 cm^−1^. The spectrometer was equipped with an iD5 attenuated total reflection (ATR) accessory having a diamond crystal, collecting 16 scans in the 400–4000 cm^−1^. Electrospun nanofibers were collected on the glass slides attached onto a rotating drum. Next, NF were separated from the glass slides. FTIR measurements were performed with three different samples of each group from three different points.

X-ray photoelectron spectroscopy (XPS) measurement was performed on non-CAP treated NF, 45 seconds CAP treated NF, non-CAP treated peptide conjugated NF, 45 seconds CAP treated peptide conjugated NF using a spectrometer (Thermo K-Alpha XPS, Thermo Fisher Scientific, West Palm Beach, FL, USA). The XPS data were recorded using monochromatic Al Kα excitation. A hemispherical electron energy analyzer (180° double focussing) with a 128- multichannel detector system was used. The analysis chamber was allowed to pump with a pressure of 2 × 10^−7^ mbar. Survey spectra and high-resolution spectra were acquired using a constant analyser with pass energies of 50 eV for single-point analysis of the surface area of each sample. X-ray beam size was 300 µm and the detector input angle was 45°. Data analysis and manipulation were performed using the Avantage XPS software with Gaussian/Lorentzian peak shapes and a Shirley/Smart type background.

To determine peptide surface coverage on NF, fluorescein isothiocyanate (FITC) attached peptide was conjugated to NF as previously described^27^. pNF, CAP treated FITC labelled GLU peptide conjugated NF (GLU-pNF), CAP treated FITC labelled ASP peptide conjugated NF (ASP-pNF) were imaged with an inverted fluorescent microscope (Olympus CKX41, Tokyo, Japan).

### Cell Seeding

Human bone marrow derived mesenchymal stem cells (hMSCs) (HMSC-AD-500, CLS cell lines Service, Lot #102, Eppelheim, Germany) was cultivated in basal medium (DMEM supplemented with 10% FBS, 100 units/mL penicillin, 100 µg/mL streptomycin, 50 µg/mL gentamicin, and 250 ng/mL fungizone). Cultures were replaced with fresh medium at 3 and 7 days (d) and cells at passage 3 were used for seeding. The edges of 13 mm circular glass coverslip covered by the NF were coated by a medical-grade silicone sealant (Dow Corning, MI) to avoid separation of the NF from coverslips. The NF were sterilized by ultraviolet (UV) radiation followed and rinsed with 70% ethanol for 30 min and washed three times with sterile PBS. After conditioning the fiber mesh in basal medium for 1 hour, each sample was seeded with 50 µL MSC cell suspension (5 × 10^4^ cells/cm^2^) in basal medium. After incubation for 24 h for cell adhesion, the medium was replaced with osteogenic medium (basal medium supplemented with 100 nM dexamethasone, 50 µg/mL ascorbic acid, 10 mM ß-glycerophosphate) and cultured in a humidified 5% CO_2_ incubator for up to 28 days. MSCs seeded on pNF and incubated in osteogenic medium were used as the negative control group.

For observation of cell morphology on surface modified NF, actin filaments and cell nuclei were stained by phalloidin and DAPI, respectively according to manufacturer’s instructions as previously described^21^ (Merck Millipore, Actin Cytoskeleton and Focal Adhesion Staining Kit, Catalog No. FAK100). Briefly, cell-seeded micro-sheets were washed twice in PBS and fixed with 4% paraformaldehyde (Sigma Aldrich, St. Louis, MO, USA) at 4 °C for 20 minutes. Next, samples were permeabilized with 0.1% Triton X-100 in PBS for 5 minutes and blocked with 1.5% bovine serum albumin (BSA) in PBS for 30 minutes. Samples were then incubated with phalloidin in PBS for 1 h at 4 °C and DAPI for 5 minutes. The stained samples were imaged with an inverted fluorescent microscope to observe cell morphology.

### Osteogenic Differentiation of hMSCs on Nanofibers

At each time point (7, 14, 21, and 28 days), cell-seeded NF were washed with PBS and lysed with 10 mM Tris supplemented with 0.2% triton in PBS. The lysed samples were used for measurement of DNA content, ALP activity and calcium content. To observe initial cellular concentration on scaffolds, DNA content was also measured at day 0. Double-stranded DNA content, ALP activity and calcium content of the samples were measured with DNA Quantification Kit (Sigma Aldrich, St. Louis, MO, USA), QuantiChrom ALP assay (Bioassay Systems, Hayward, CA, USA) and QuantiChrom Calcium Assay (Bioassay Systems, Hayward, CA, USA), respectively according to manufacturer’s instructions as previously described^27^.

Briefly, bisBenzimide H 33258 Solution were prepared and added on lysed samples in a 96-well plate. Fluorescence (excited at a wavelength of 360 nm) was measured using a spectrophotometer (BioTek, Winooski, VT, USA) at an emission wavelength of 460 nm, at ambient temperature. ALP activity was evaluated by p-nitrophenylphosphate (*p*NPP) at 405 nm in alkaline solution using ALP kit. First, 50 μL of lysed sample to 200 μL total reaction volume were used to initiate the reaction by the addition of assay buffer, 5 mM magnesium acetate, and 10 mM *p*NPP in 96-well plate. Optical density (OD) in 405 nm was measured at initial time (t = 0) and after 4 min (t = 4 min) on multi plate reader (BioTek, Winooski, VT, USA). Calcium content of the NF was measured by adding 50 μL of the suspension to 150 μL of the working solution. After incubation, OD at 612 nm was correlated to the equivalent amount of Ca^2+^ using a calibration curve plotted with reference calcium solutions. Total mineralized deposit of each sample was determined from the measured calcium content at each time-point. The measured ALP activities and calcium contents were normalized to cell numbers by dividing to DNA contents at each time point.

### Quantitative real-time PCR analysis

At each time point (7, 14, 21 and 28 days), total cellular RNA was isolated using Blood/Cell Total RNA Mini Kit (Geneaid, Sijhih City, Taiwan). Then, the extracted purified RNA was subjected to cDNA conversion using M-MuLV First Strand cDNA Synthesis Kit (Biomatik, Ontario, Canada). The cDNA obtained was subjected to Step One Plus Real-time PCR system (Applied Biosystems, Foster City, USA) amplification with appropriate gene-specific primers. Forward and reverse primers for RT-qPCR, shown in Table 1, including osteocalcin (OCN), osteopontin (OPN), Collagen type I (Col I) and glyceraldehyde 3-phosphate dehydrogenase (GAPDH) were purchased from Sentegen Biotechnology (Ankara, TURKEY) and used to evaluate gene expression^36^. The differential expression of genes Osteopontin (OP), Osteocalcin (OC) and collagen type I (COL-1) was quantified by by StepOne Software v2.3 and Ct values were classified by 2^(−ΔΔCt)^ method described elsewhere^37,38^. Every group was experimented in qPCR as doublet and repeated as triplicate (n = 6).

**Table 1:** Forward and reverse primers. Collagen Type I (Col-1), Osteopontin (OP), Osteocalcin (OC), and glyceraldehyde 3-phosphate dehydrogenase (GAPDH; housekeeping gene) (GAPDH) used to asses hMSCs differentiation in qRT-PCR amplification.

Genes	Forward Primer	Reverse Primer
Collagen type I	5′-TGA CGA GAC CAA GAA CTG-3′	5′-TCA GCC TTA GAC GCC TCA AT-3′
Osteopontin	5′-ATG AGA TTG GCA GTG ATT-3′	5′-TTC AAT CAG AAA CTG GAA-3′
Osteocalcin	5′-TGT GAG CTC AAT CCG GAC TGT-3′	5′-CCG ATA GGC CTC CTG AAG C-3′
GAPDH	5′-AAC AGC GAC ACC CAC TCC TC-3′	5′-CAT ACC AGG AAA TGA GCT TGA CAA-3′

### Immunofluorescent staining

For immunofluorescence staining, cell-seeded NFs were rinsed twice in PBS and fixed with 4% paraformaldehyde (Sigma Aldrich, St. Louis, MO, USA) at 4 °C for 30 minutes. Next, samples were immersed with 0.1% Triton X-100 in PBS for 1 hour and blocked with 1.5% Bovine Serum Albumin (BSA) in PBS for 2 hours. Then, samples were incubated with primary antibodies in PBS containing 1% BSA overnight at 4 °C according to manufacturer’s instructions. Primary antibodies (Santa Cruz Biotechnology Inc., Santa Cruz, California, USA) included mouse monoclonal antibody against COL I (Cat. No. sc-59772; 1:50), mouse monoclonal against OP antibody (Cat. No. sc-21742; 1:50), and mouse monoclonal antibody against OC (Cat. No. sc-365797; 1:500). Fluorescence secondary antibodies (Santa Cruz Biotechnology Inc., Santa Cruz, California, USA) included m-IgG kappa BP-FITC (Cat. No. sc-516140; 1:200) and m-IgG kappa BP-PE (Cat. No. sc-21742; 1:200). Secondary antibodies were diluted with 1% BSA before use. It should be noted that each sample was stained with 4,6-diamidino- 2-phenylindole (DAPI, Sigma Aldrich, St. Louis, MO, USA) to image the cell nuclei and one of the antibodies for COL-I, OC, and OP. Secondary antibodies without the primaries were used as negative controls. The expression pattern of COL-I, OP, and OC with same exposure time and light intensity were characterized via capturing images using an inverted fluorescence microscope (Olympus CKX41, Tokyo, Japan).

### Statistical analysis

All data are statistically analyzed by two-way ANOVA (SPSS 12.0, SPSS GmbH, Germany) and the Student-Newman-Keuls method as a post hoc test. Significant differences between groups were determined using p values less than 0.05. (*p < 0.05, **p < 0.01, ***p < 0.001).

## Results and Discussion

### Characterization of CAP treated GLU and ASP conjugated Nanofibers

**Figure 1:**
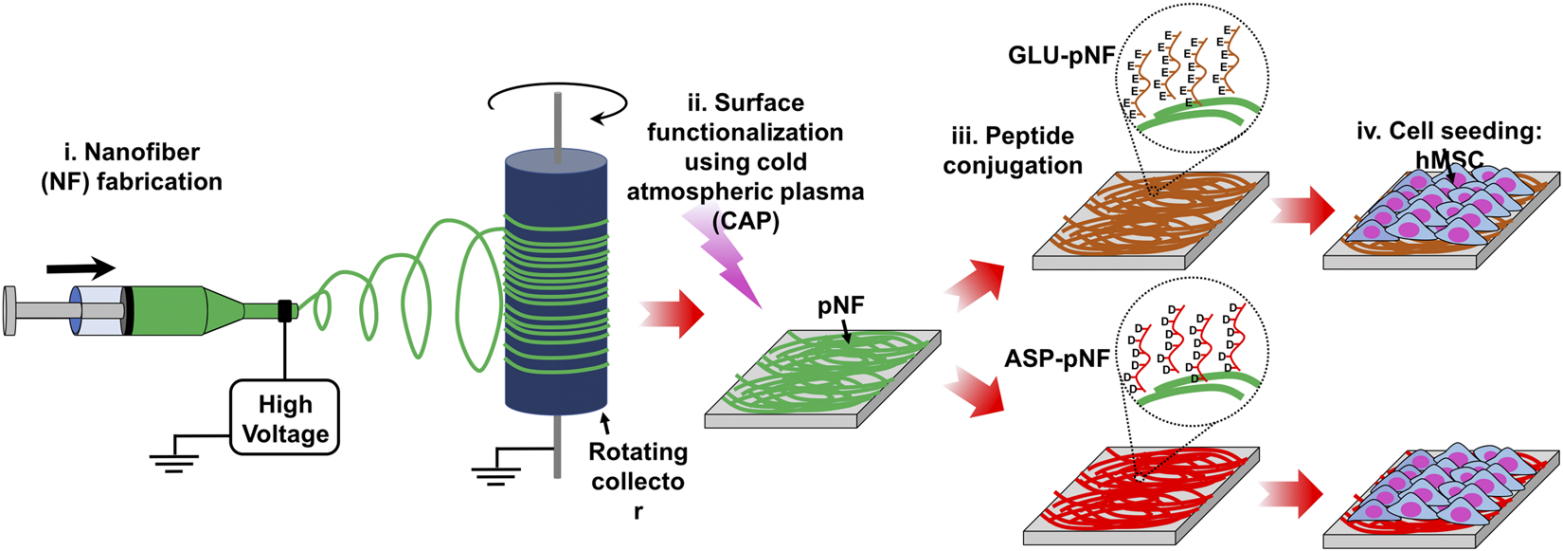
Schematic diagram of cold atmospheric plasma (CAP) assisted conjugation of Glutamic (Glu) and Aspartic (Asp) acid templated peptides on PLGA nanofibers (NF). The steps of the illustration: nanofiber fabrication (i), surface functionalization using cold atmospheric plasma (pNF, ii), peptide conjugation (GLU-pNF, ASP-pNF, iii), hMSC seeding and osteogenic differentiation on control (pNF), GLU-pNF, and ASP-pNF (iv).

**Figure 2:**
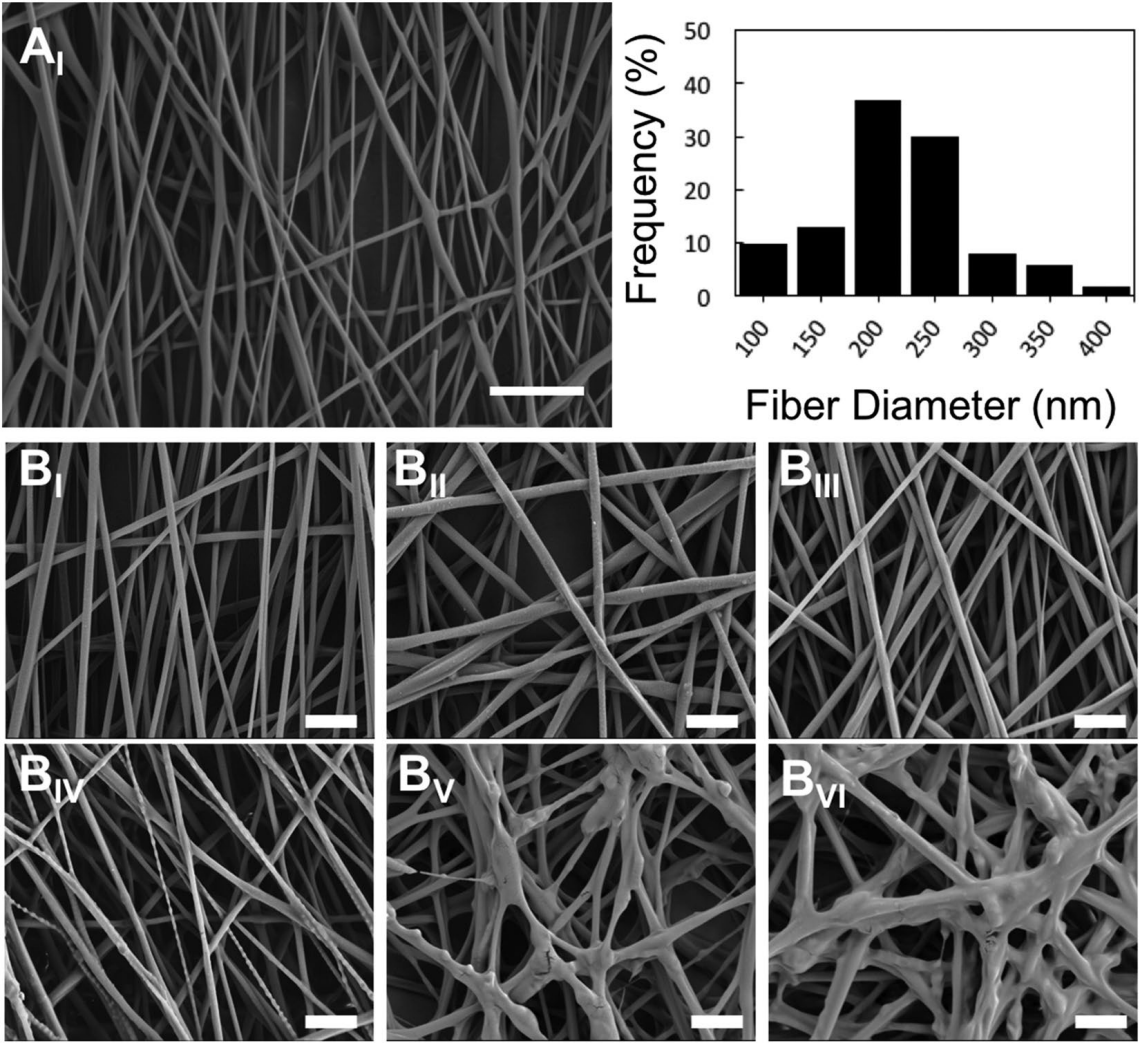
Scanning electron microscopy images of PLGA nanofibers (NF) (**A**_**I**_) (Scale bar represents 200 µm), histogram showing nanofiber diameters distribution (**A**_**II**_), non-CAP treated (**B**_**I**_), 15 (**B**_**II**_), 30 (**B**_**III**_), 45 (**B**_**IV**_), 60 (**B**_**V**_), and 90 (**B**_**VI**_) seconds CAP treated PLGA NF (Scale bar represents 2 µm).

ASP and GLU peptides were conjugated on to the surface of CAP treated NF using EDC/NHS as coupling agents and further osteogenic differentiation of hMSCs were conducted on experimental groups of pNF (control), GLU-pNF, and ASP-pNF, as depicted in Fig. 1. The amide reaction between amino and carboxyl groups is expected to be difficult as basic amino groups deprotonates carboxyl group forming a highly unreactive carboxylate. Usually some coupling reagents are used to activate the carboxyl group to improve the yield of a carboxylic acid/amine coupling. Due to simplicity and high efficiency, EDC/NHS are the most widely used coupling reagents. The reaction can also take place in aqueous solutions, which helps avoiding the toxic effects of organic solvents. Basically, EDC activates the carboxylic groups on NF, forming an unstable o-acylisourea intermediate in MES buffer known to be a suitable carbodiimide reaction buffer. Then, NHS replaces EDC, forming an NHS ester which is a more stable reactive than o-acylisourea intermediate and still susceptible to attack by the N of a primary amine, thus leading to an efficient conjugation at physiological pH. An SEM photomicrograph of PGLA NF produced by electrospinning is given in Fig. 2A_I_. NF diameter distribution histogram was given in Fig. 2A_II_. Diameter of the resulting NFs used throughout the study ranged from 100 to 400 nm and the mean diameter was calculated to be 246 ± 24 nm. Next, the impact of surface treatment with CAP on the morphology of NF was investigated. In summary, CAP was used to introduce carboxyl (-COOH) groups to the surface of NF for conjugation with bone ECM mimetic peptides. The surface of the NF was treated for 15, 30, 45, 60 and 90 seconds and the results were compared to non-CAP treated NF. As shown in Fig. 2B_II–VI_, the morphology of the NF did not change drastically up to 45 s treatment. However, longer CAP treatment (60 and 90 s) caused a significant change in the morphology of NF as they started to join together. Our results were consistent with that of Chen *et al*. who reported 40 s of CAP treatment did not change the morphology of poly(L-lactic acid) (PLLA) NF and drastically increased carboxyl groups on NF surface^39^. Effect of plasma treatment on the nanofibers is mainly governed by oxidation process^40^. Moreover, various effects of plasma such as antimicrobial effect and enhancing hydrophilicity of surfaces are treatment time dependent and tend to increase with plasma treatment time^21,41^. Even though, further studies are needed and it is hard to draw a certain conclusion, extensive oxidation in consequence of increasing plasma treatment time is more likely responsible for the morphology change of nanofibers.

**Figure 3:**
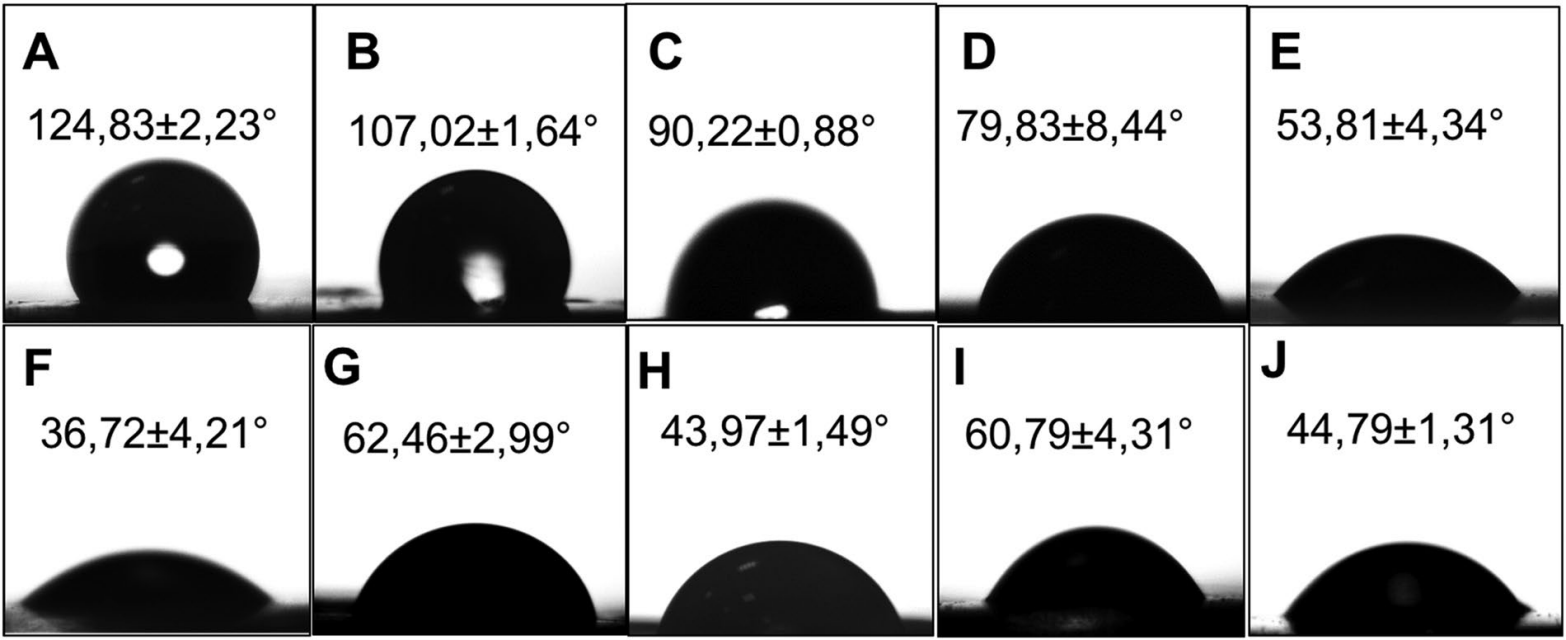
Water contact angles of surface modified nanofibers. Non-CAP treated (**A**), 15 s (**B**), 30 s (**C**), 45 s (**D**), 60 s (**E**) and 90 s CAP treated (**F**), non-CAP treated GLU peptide conjugated NF (GLU-NF) (**G**), 45 s CAP treated GLU peptide conjugated (GLU-pNF) (**H**), non-CAP treated ASP peptide conjugated (ASP-NF) (**I**) and 45 s CAP treated ASP peptide conjugated (ASP-pNF) (**J**).

The impact of CAP treatment for 15, 30, 45, 60 and 90 s on hydrophilicity of the NF was also demonstrated using water contact angle (θ) measurement. Water contact angle on NF were determined based on the images taken right after 10 µL of deionized water was dripped to the surface and they were compared to that of non-CAP treated NF. As demonstrated in Fig. 3A–F that the contact angle (θ) of non-CAP treated NF was 124.83 ± 2.23° and dropped down to 36.72 ± 4.21° after 90 s CAP treatment. These results were in agreement with that of Dolci *et al*. who observed significant change on water contact angle and hydrophilicity of PLLA NF after CAP treatment, due to the increased carboxyl groups (-COOH)^42^. In the present study, air plasma was generated and is known to have oxidative effect due to presence of reactive oxygen species^40^. As shown and supported by FTIR and XPS analyses, plasma treatment cleaves C-C and C=C bonds on the backbone of the nanofiber, and ROS created in the plasma are bounded to free ends of the carbon atoms exposed by the cleavage of single and double bonds which consequently leads to formation of –COOH groups on the surface of nanofibers. To be able to enhance peptide conjugation, it is important to maximize and reproduce the introduction of -COOH groups to the NF without losing their integrity. For that purpose, 45 seconds was chosen as the optimum CAP treatment time duration in the following experiments. Accordingly, the impact of the GLU and ASP peptide conjugation on the water contact angle (θ) of non-CAP treated (GLU-NF and ASP-NF) and 45 s CAP treated NF (GLU-pNF and ASP-pNF) were also shown (Fig. 3G–J). The water contact angles (θ) of GLU-NF (Fig. 3G), GLU-pNF (Fig. 3H), ASP-NF (Fig. 3I) and ASP-pNF (Fig. 3J) were measured as 62.46 ± 2.99°, 43.97 ± 1.49°, 60.79 ± 4.31° and 44.79 ± 1.31°, respectively. The water contact angles of GLU-NF and ASP-NF were measured to be lower than non-CAP treated NF (Fig. 3A), which is normal considering the fact that both GLU and ASP peptides are negatively charged at pH 7.0. The impact of CAP treatment on hydrophilicity of peptide modified scaffolds was evident in GLU-pNF and ASP-pNF because the water contact angle was considerably lower than that of non-CAP treated NF.

**Figure 4:**
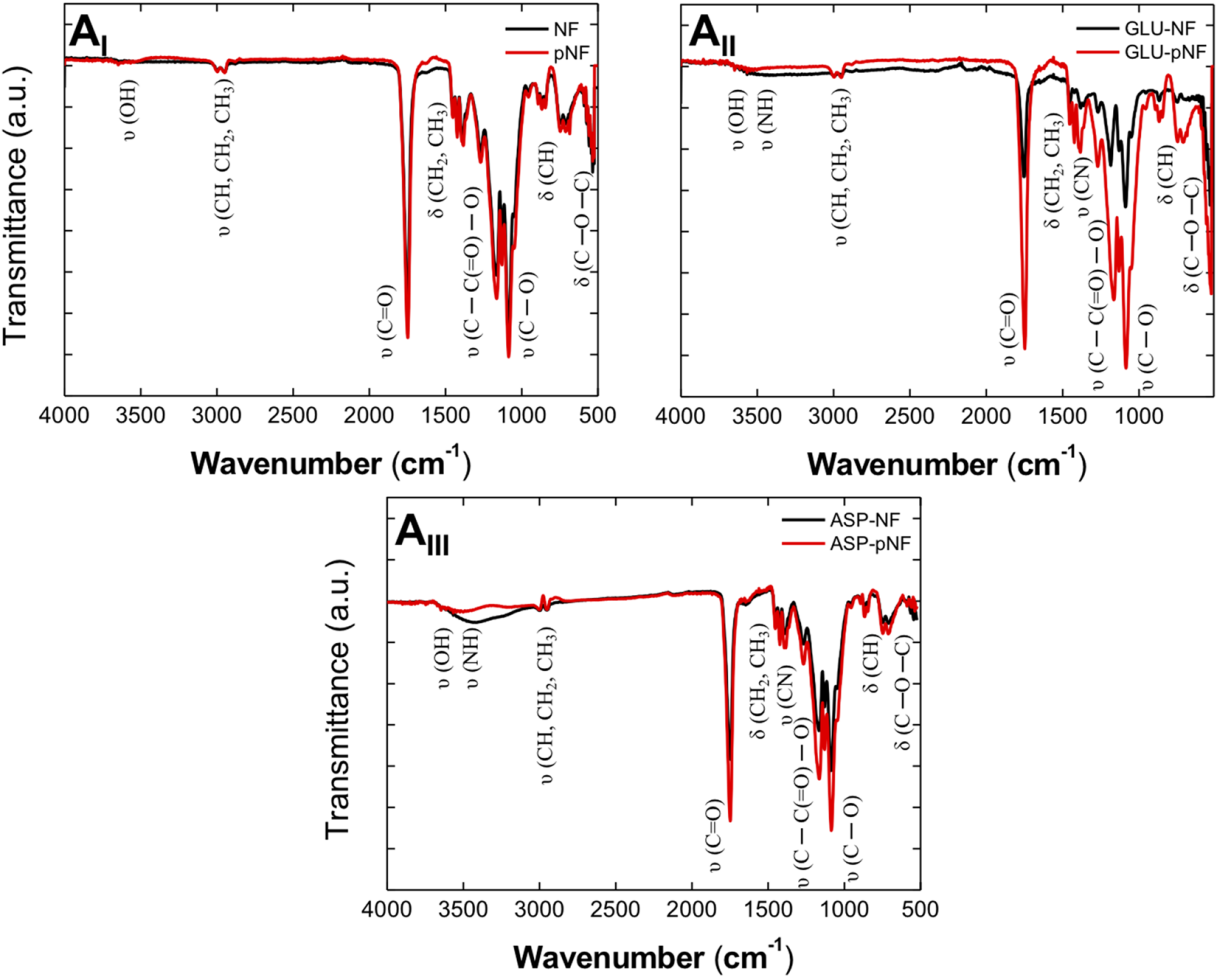
FTIR spectra for PLGA nanofibers (NF): before and after CAP treatment (NF and pNF). (**A**_**I**_) GLU peptide conjugated (GLU-NF) and CAP treated GLU peptide conjugated (GLU-pNF). (**A**_**II**_) ASP peptide conjugated (ASP-NF) and CAP treated ASP peptide conjugated (ASP-pNF). (**A**_**III**_) (CAP treatment time is 45 seconds).

ATR-FTIR spectroscopy was used to further confirm the surface group functionalization through CAP treatment. Figure 4 shows the FTIR spectra of the pNF (Fig. 4A_I_) and/or peptide conjugation (Fig. 4A_II,III_). In general, the broad bands appeared at around 3600 and 3000 cm^−1^ due to the presence of hydroxyl and alkyl groups. The sharp bands at around 1750 and 1080 cm^−1^ were assigned to the C=O stretch and the C-O stretch of the PLGA polymer^43,44^.The bands occurring at the range of 1420–1400 cm^−1^ and 900–690 cm^−1^ were attributed to the CH_2_, CH_3_, and CH bending vibrations. The strong stretching bands due to the asymmetric and symmetric C-C(=O) -O vibrations were observed between 1200–1150 cm^−1^ ^45^. There are also C-O-C bending bands appearing at the 560–520 cm^−1^ region. In the case of peptide conjugated NF, a change in the absorption bands at 3600–3000 cm^−1^ and 1460–1240 cm^−1^ was observed by the effect of nitrogen introduction caused by peptide conjugation (see FTIR spectra for GLU and ASP in Fig. S5, Supporting Information). Figure 4A_I_ shows that the transmittance intensity of the NF increased upon exposure to CAP treatment indicating the inclusion of oxygen-containing groups such as carbonyl and carboxyl groups on the NF surface^46,47^. The same trend was more predominantly observed for peptide conjugated (GLU-NF, ASP-NF) and CAP treated peptide conjugated (GLU-pNF, ASP-pNF) NF (Fig. 4A_II,III_). Particularly, the intensity of C-C(=O)-O, C-O, and C-O-C bands became large and broadened with CAP treatment. Overall, this functionalization clearly shows the positive impact of CAP treatment on conjugation short peptides with NF.

**Figure 5:**
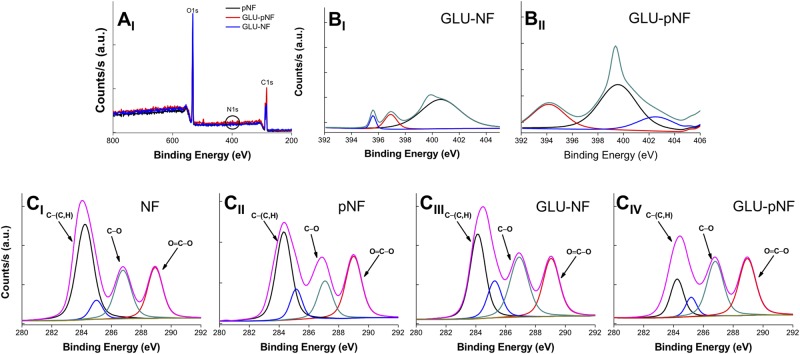
XPS wide scan (**A**_**I**_) of CAP treated NF (pNF), non-CAP treated GLU peptide conjugated NF (GLU-NF), and CAP treated GLU peptide conjugated NF (GLU-pNF). N1s high-resolution spectra (**B**_**I**_,**B**_**II**_) of the GLU-NF and GLU-pNF. C1s high-resolution spectra (**C**_**I–IV**_) of NF, pNF, GLU-NF, and GLU-pNF. (CAP treatment time is 45 seconds).

XPS analysis was also performed to investigate the changes in chemical composition of the PLGA nanofiber surfaces after CAP treatment and/or peptide conjugation. The elemental composition of the nanofiber surfaces was given in Table 2. Not surprisingly, the oxygen atomic concentration of the CAP treated NF is higher than that of the non-treated NF, indicating the formation of oxygen-rich functional groups with CAP treatment. This observation also suggests that the enhanced surface modification of pNF by GLU and ASP peptides. Since the similarity in chemical structures of GLU and ASP, a slight difference in conjugation and/or CAP treatment was observed at peptide conjugated NF. Figure 5 shows XPS wide scan (Fig. 5A_I_), N1s (Fig. 5B_I_,B_II_), and C1s high-resolution spectra (Fig. 5C_I–IV_) of NF with different surface chemistries. The wide survey of the NF contained peaks arising from the backbone of PLGA, namely carbon (284 eV) and oxygen (532 eV); and nitrogen (399 eV) that corresponded to GLU indicating the binding of the peptide on the NF surface. There are no N1s signals in NF and pNF, while N1s signals were observed for peptide conjugated and CAP treated peptide conjugated NF (GLU-NF, ASP-NF, GLU-pNF, and ASP-pNF). The enlarged N1s XPS spectra show the effect of CAP treatment on the peptide conjugation. N1s signals can be deconvoluted into the three components at approximately 401, 397, and 396 eV corresponding to N-O, N=C, and N-C bonds^46^. C1s high-resolution XPS spectra showed main signals observed at 289, 287, 285, and 284 eV which arise from three different C environments, namely O=C-O, C-O, C-(C, H), respectively^48^. GLU and ASP both have the same carbon environments^49^ (see also C1 spectra of ASP-NF and ASP-pNF in Figure S6, Supporting Information). CAP treatment changes these atomic compositions, particularly, the intensity of O=C-O and C-O bonds significantly increased compared to that of non-treated PLGA nanofibers (NF). XPS analysis proved that the number of polar groups on PLGA NF surface increased after plasma treatment and the enhanced surface hydrophilicity enabled peptide conjugation. This result seems to be in line with the FTIR measurements.

**Table 2:** Atomic composition and relative peak intensities of PLGA nanofibers.

Nanofibers	Atomic Composition (%)			Relative Peak Intensity (%)		
	C	O	N	C-C	C-O	O-C=O
NF	62.2	37.8	—	79.3	10.5	10.2
pNF	58.5	41.5	—	74.5	13.3	12.2
GLU-NF	61.3	36.8	1.9	75.4	13.2	11.4
GLU-pNF	51.9	47.2	0.9	71.4	15.0	13.6
ASP-NF	62.3	36.0	1.7	75.6	13.0	11.4
ASP-pNF	50.9	48.3	0.8	70.6	15.4	14.0

**Figure 6:**
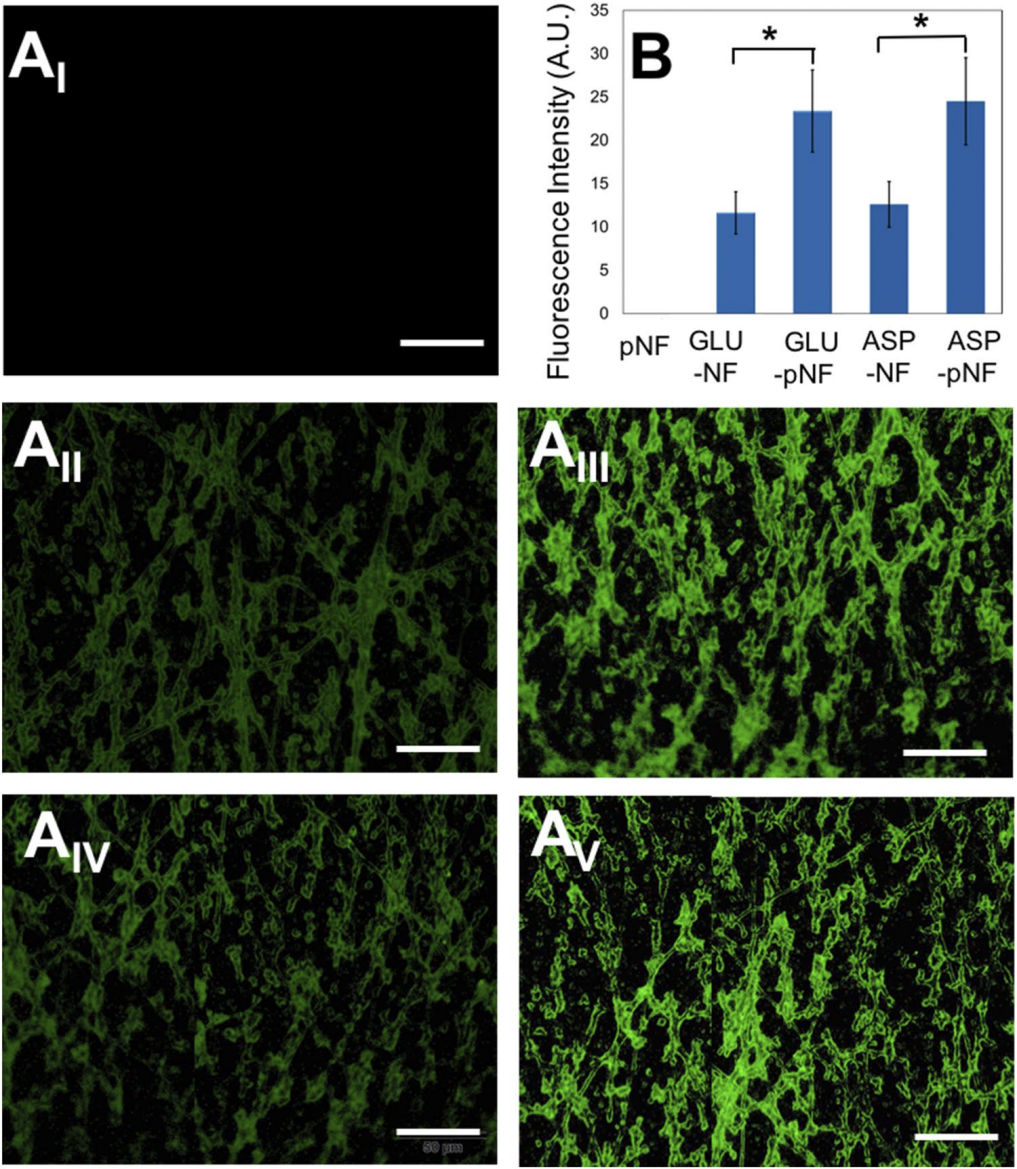
Fluorescent microscopy images of CAP treated pNF (**A**_**I**_), non-CAP treated fluorescein isothiocyanate (FITC) labeled glutamic acid peptide conjugated NF (GLU-NF). (**A**_**II**_) CAP treated FITC labeled glutamic acid peptide conjugated NF (GLU-pNF) (**A**_**III**_), non-CAP treated fluorescein isothiocyanate (FITC) labeled aspartic acid peptide conjugated NF (ASP-NF). (**A**_**IV**_) CAP treated FITC labeled aspartic acid peptide conjugated NF (ASP-pNF) (**A**_**V**_) (Scale bar represents 50 µm). Fluorescence intensity (A.U.) of pNF, GLU-NF, and GLU-pNF (**B**).

The efficacy of CAP treatment on peptide conjugation was also characterized via observing FITC intensity by using fluorescence microscope. FITC labeled GLU peptides were used to show the NF surface coverage with peptides. Briefly, FITC labelled GLU peptides were conjugated with the electrospun NF following 45 s CAP treatment. The images were then compared to those of NF, GLU-NF, GLU-pNF, ASP-NF, and ASP-pNF. The mean (five different samples) fluorescence intensity of each image was also determined using IMAGEJ for further comparison. As shown in Fig. 6A,B, the highest fluorescence intensity was observed with GLU-pNF and ASP-pNF. pNF only were used as negative control and as expected no fluorescence was observed, which confirms that the fluorescence observed in the other two images came from the label FITC (see grayscale images of GLU-NF, GLU-pNF, ASP-NF, and ASP-pNF in Fig. S4, Supporting Information). In our previous study, we indicated that CAP treatment on titanium discs increased the hydroxyl groups on the surface and that also sharply increased the FITC labelled RGD peptide conjugation^21^. Introduction of high number of carboxyl group to the surface with CAP, facilitated most of the surface to be covered with peptides. In addition, fluorescence intensity of GLU-pNF and ASP-pNF groups showed significantly higher intensity compared to GLU-NF and ASP-pNF, respectively. It is speculated that available carboxyl groups even before CAP treatment facilitated peptide conjugation. Increasing carboxyl groups on NF surface via CAP treatment drastically increased peptide conjugation. Based on these findings, appropriate duration of CAP treatment could be used as an intermediate step to increase peptide conjugation to polymeric NF.

### Differentiation of hMSCs on CAP treated GLU and ASP conjugated NF

**Figure 7:**
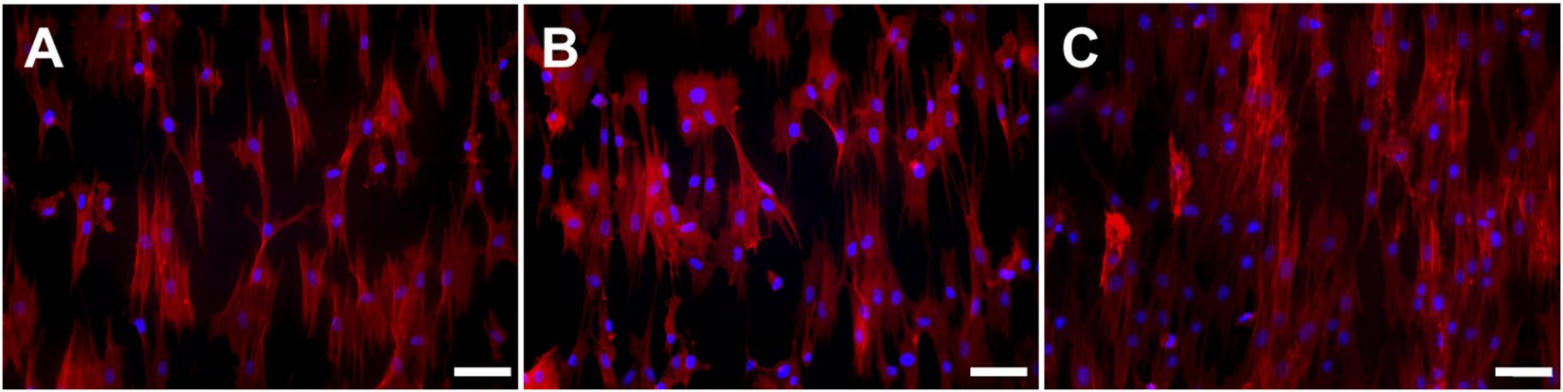
Morphology of human marrow stromal cells (hMSCs) seeded on CAP treated pNF (**A**), GLU-pNF (**B**) and ASP-pNF (**C**) after culturing in osteogenic media for 7 days. In the images, cell nuclei and cytoskeletal actin are stained with 4,6-diamidino-2-phenylindole (DAPI; blue) and phalloidin (red) (Scale bar represents 50 µm).

Osteogenic differentiation capability of hMSCs over pNF conjugated with GLU and ASP peptides was assessed, respectively. First the morphology of the cells on these surface modified NFs was examined after culturing in osteogenic medium for 7 days using a fluorescence microscopy. The actin cytoskeleton of cells was stained using phalloidin and nuclei with DAPI prior to fluorescence imaging. Resulting images show that hMSCs cells successfully attached and spread on pNF, GLU-pNF, and ASP-pNF (Fig. 7). Additionally, we previously reported that there is no difference in cell morphology of MSCs when seeded on neat PLGA NF, Glu enriched peptide conjugated NF and mineralized glutamic acid enriched peptide conjugated PLGA NF and cultured in osteogenic media^27^. Due to the fact that osteogenic media contains molecules that induce osteogenic differentiation, cell morphology on both groups resembles osteoblast.

**Figure 8:**
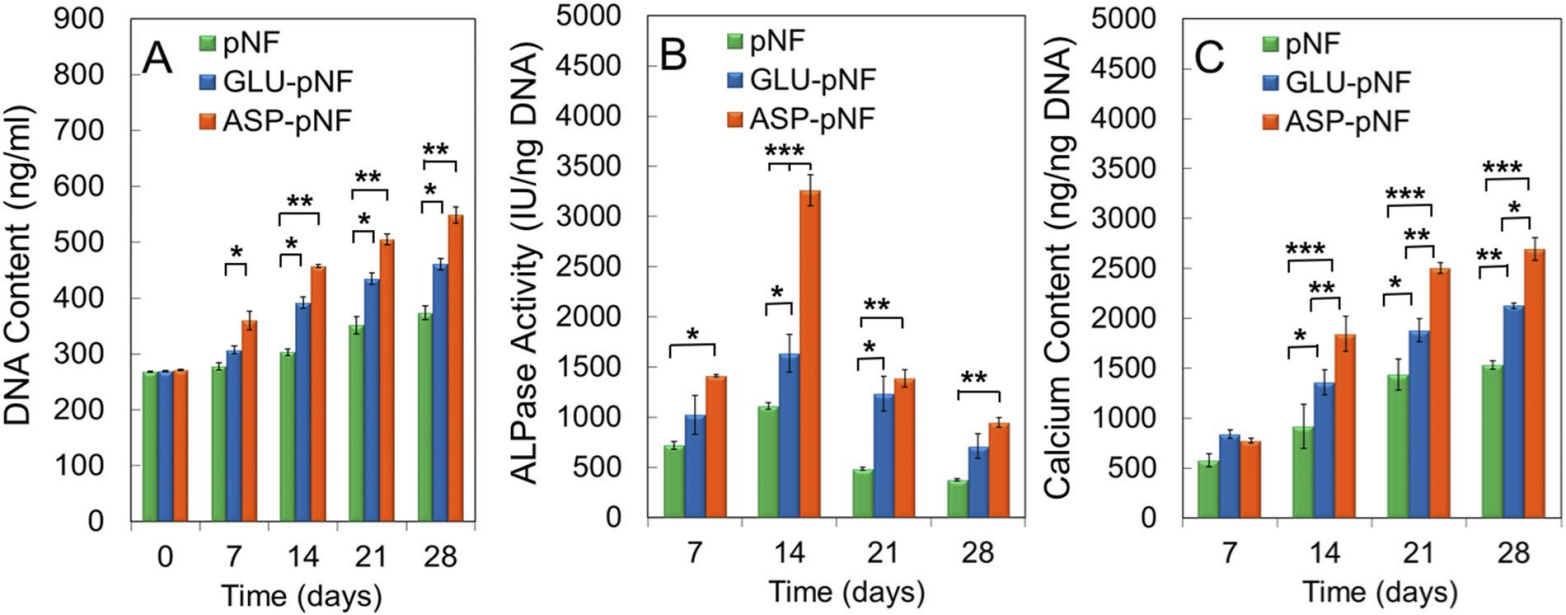
DNA content (**A**) ALP activity (**B**) and calcium content (**C**) of human marrow stromal cells (hMSCs) seeded on CAP treated pNF, GLU-pNF, and ASP-pNF and incubated in osteogenic medium for up to 28 days. Error bars represent mean ± SE (n = 5) [significant differences were determined by one-way ANOVA [Newman–Keuls multiple comparison test, (*p\0.05, **p\0.01, ***p\0.001)].

Differentiation of hMSCs into osteoblastic lineage is a complex process, which includes hMSCs adhesion, proliferation, differentiation, maturation, and mineralization^50^. Osteogenic differentiation can be guided through bone ECM mimetic peptide sequences and thus, bioinert synthetic scaffolds may gain transformed to osteoinductive capability^50^. The most crucial parameters that could be used to evaluate osteogenic differentiation are sufficient cell growth, ALP activity, calcium deposition and expression of osteogenic markers^27,51–53^. In this regard, osteogenic differentiation of hMSCs on surface modified NF was first quantified by monitoring cell number, ALP activity and calcium deposition over 28 days. The cell number was determined based on DNA content. At this stage, hMSCs were seeded and incubated on pNF, GLU-pNF, and ASP-pNF for 7, 14, 21 and 28 days and at the end of each culture time period, DNA content of cells was measured. Results revealed that ASP-pNF facilitated the proliferation of hMSCs significantly compared to GLU-pNF and pNF throughout the 28 days of incubation period (Fig. 8A). This result suggest that ASP induces cell proliferation more than GLU, which can be related to the presence and more influential role of ASP in integrin binding motif^54^. As mentioned earlier, cell proliferation is the initial and key step for bone regeneration and directly coordinate the following maturation and mineralization stages^55^. Our results are consistent with an early report that showed enhanced MC3T3-E1 pre-osteoblasts proliferation when seeded on bone tissue ECM mimetic peptide conjugated synthetic scaffolds^56^.

ALP activity measurement was carried out using the time-dependent pNPP formation in alkaline solution. As can be seen in Fig. 8B, the ALP activity was the highest in the case of ASP-pNF at all time points when compared to other groups suggesting that cells did not receive as much osteogenic induction in pNF and GLU-pNF groups. Consistent with previous results, ALP activity appeared to rise from day 7 to 14, and then started to decrease with longer period of incubation with mineralization^27,57,58^. At day 14, ALP activity (IU/ng DNA) of ASP-pNF was significantly higher (3261.9 ± 152.9) then those of pNF (1112.5 ± 33.4) and GLU-pNF (1636.8 ± 186.5). It should also be noted that ALP activity of cells on GLU-pNF was higher than those on pNF, indicating the positive impact of GLU on osteogenic differentiation. Calcium deposition is an indicative of maturation stage and for that hMSCs are expected to increase their calcium content^59^. hMSCs seeded groups of pNF, GLU-pNF, and ASP-pNF showed a consistent elevation of calcium content (ng Ca/ng DNA) throughout the incubation time and reached to 1531.8 ± 42.7, 2127.1 ± 26.1, and 2695.5 ± 111.7, respectively. Results revealed that calcium content on ASP-pNF was significantly higher than pNF and GLU-pNF at all time points and peak at day 28 (Fig. 8C). Cells seeded on pNF only had the lowest calcium content, suggesting that cells did not mature as much compared to GLU-pNF and ASP-pNF. These results suggest that GLU and ASP conjugation sharply enhances mineralization of NF even at the early stages of culture and increased calcium phosphate content significantly accelerates osteogenic differentiation of hMSCs. As previously reported by our group and elsewhere, mineralized NF significantly promotes osteogenic differentiation of hMSCs^27,60,61^. In a recent study, Sun *et al*. indicated that seven ASP sequences implemented bone morphogenic protein (BMP) mimetic peptide [P28(D7)] significantly elevated ALP activity and Ca^2+^ content of MC3T3-E1 pre-osteoblasts. It was also demonstrated that ASP sequence had higher interaction with bone forming cells and minerals in osteogenic media compared to seven GLU sequence BMP mimetic peptide [P28(E7)]^62^. Additionally, they also reported that P28 peptide enriched nano-hydroxyapatite scaffolds significantly accelerated bone regeneration in critical-sized rat cranial defects at 6 and 12 weeks post-implantation compared to scaffolds lacking P28^63^.

**Figure 9:**
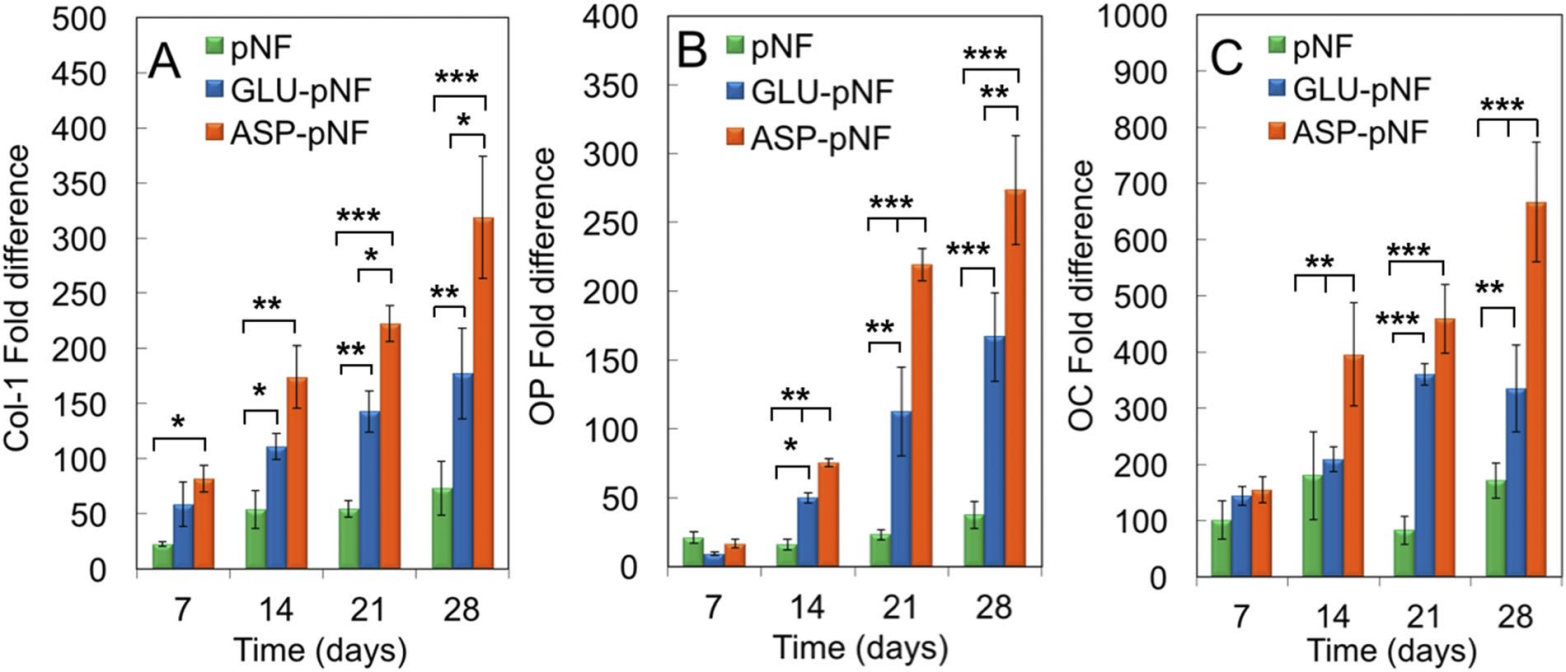
The mRNA expression levels (as fold difference) of type 1 collagen (Col-I) (**A**) osteopontin (OP) (**B**) and osteocalcin (OC) (**C**) for hMSCs seeded on CAP treated pNF, GLU-pNF, and ASP-pNF and incubated in osteogenic medium for up to 28 days. Error bars represent mean ± SE (n = 5) [significant differences were determined by one-way ANOVA [Newman–Keuls multiple comparison test, (*p\0.05, **p\0.01, ***p\0.001)].

Osteogenic differentiation was also evaluated with respect to expression of osteogenic markers including collagen type I (COL-I), osteopontin (OP) and osteocalcin (OC) using real-time PCR. Briefly, COL-I mineralizes in the presence of calcium ions into calcium phosphate which makes up roughly 70% of bone matrix, whereas OPN and OCN are extracellular matrix proteins and they mediate nucleation and stabilization of calcium phosphate crystals^50,59^. The expressions of all these markers are expected to rise throughout the 28 days of incubation considering their crucial role in bone crystallization. In line with the results obtained so far, ASP-pNF significantly induced expression of these markers as compared to pNF and GLU-pNF (Fig. 9A–C). The expression of all the markers on pNF only was the lowest, indicating the positive impact of the two peptides for bone mineralization. Importantly, it was indicated that negatively charged GLU and ASP peptides act as a calcium ion nucleation points in collagen NF during biomineralization and significantly increased the interaction of the NF with positively charged calcium ions^64^. Ca^2+^ ions can bind to aminoacids with varying affinity depending on pH. Protonation of carboxylate group weakens the binding and thus, increasing the pH results in higher affinity. It has been previously reported that Asp binds (Ka = 7.0 ± 0.9 L/mol) Ca++ ions better than Glu (Ka = 3.0 ± 0.8 L/mol) at a pH around 7^65^. Therefore, it is possible that Asp stabilizes CaP better than glutamate resulting in higher ALP activity, Ca content as well as higher expression of osteogenic gene markers on NF at neutral pH^64^. Considering the inductive effect of mineralization on osteogenic differentiation, this might be one of the reasons for higher level osteogenic gene marker expression with ASP-pNF than Glu-pNF.

**Figure 10:**
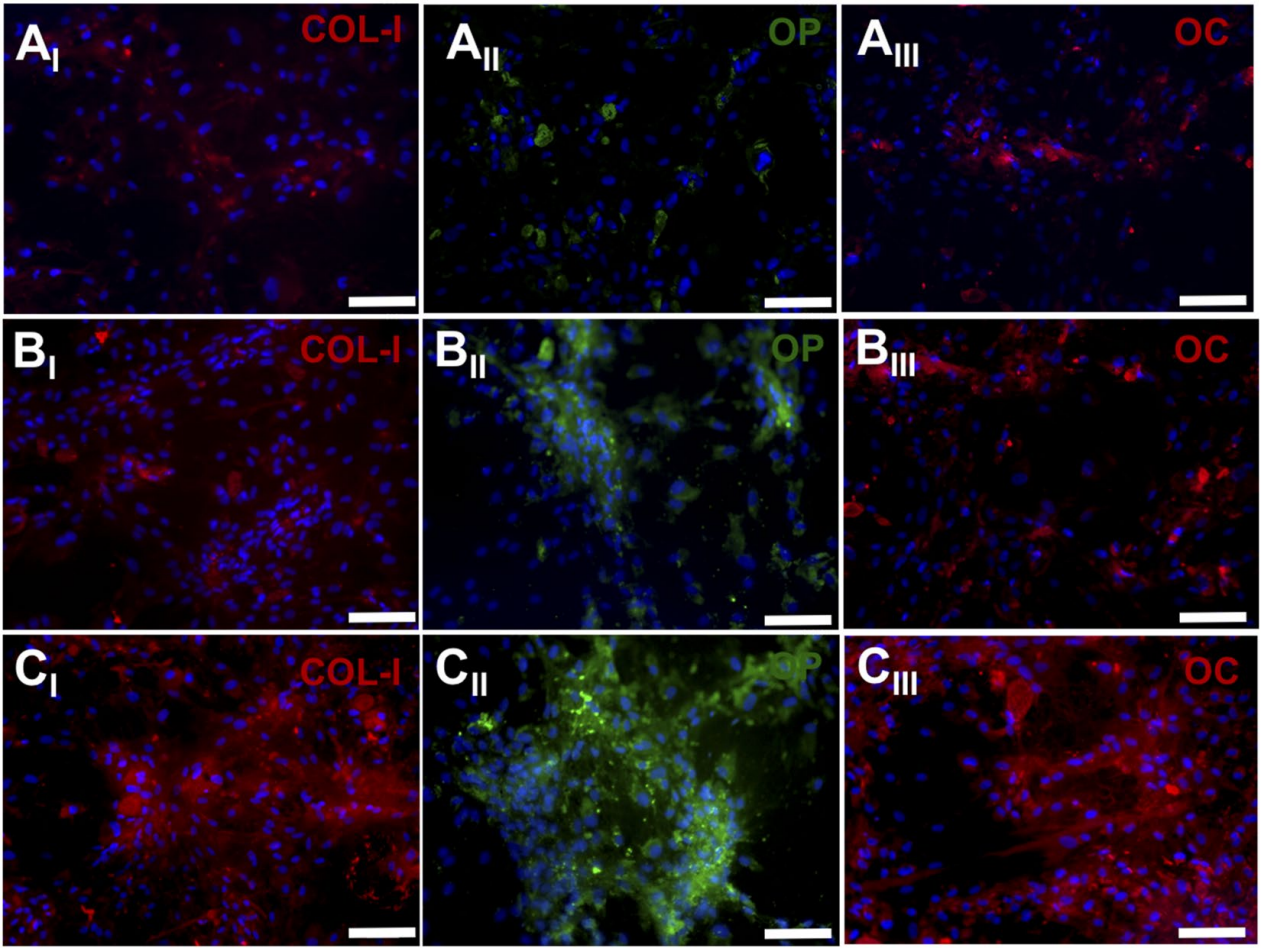
Expression pattern of osteogenic markers type 1 collagen (red, first column), osteopontin (OP) (green, second column) and t osteocalcin (OC) (red, third column) for hMSCs seeded on CAP treated pNF (**A**_**I–III**_), GLU-pNF (**B**_**I–III**_), and ASP-pNF (**C**_**I–III**_) after 28 days’ incubation in osteogenic medium. Cell nuclei in the images are stained with 4′,6-diamidino-2-phenylindole (DAPI; blue) (Scale bar represents 100 µm).

Immunofluorescence staining of differentiated hMSCs also confirmed these results. Briefly, cells were incubated in osteogenic medium for 28 days and then chemically fixed for immunostaining. Lastly, proteins were stained using first primary antibodies against COL-I (red), OP (green) and OC (red) and then labelled with secondary antibodies to be visualized using an inverted fluorescence microscopy. As demonstrated in Fig. 10, the expression of these osteogenic maturation related proteins was drastically higher for hMSCs on ASP-pNF than those on pNF and GLU-pNF, respectively. In consistent with real-time PCR results, cells on GLU conjugated NF showed an elevated expression of these markers compared to those on pNF, highlighting the osteogenic inductive effect of GLU. In our previous study, we demonstrated that two GLU sequence including ECM mimetic peptide facilitated mineralization process and significantly increased gene expression and secretion of COL-I, OC, and OP^27^. Results so far have demonstrated that the presence of the two peptides (ASP and GLU) induced osteogenic significantly when compared to only CAP treatment. The two peptides seemed to show different osteogenic inductive effect with ASP being the highest.

## Conclusion

In summary, biomimetic ASP and GLU templated peptides were successfully conjugated with electrospun NF. We found that application of CAP on the surface of NF increases hydrophilicity and the number of carboxylic groups, which could effectively enhance the binding sites for biomimetic GLU and ASP templated peptides. Conjugating GLU and ASP peptides improved the osteoinductive capacity of the NF. Moreover, ASP templated peptides sharply increased ALP activity, calcium content, and expression of key osteogenic markers of COL-1, OP, and OC compared to pNF and GLU-pNF. It was further depicted that ASP sequences are the major fragments that influence mineralization and osteogenic differentiation in non-collagenous proteins of bone extracellular matrix. Even though there are some studies showing the individual osteoinductive capacity of ASP and GLU templated peptides when conjugated with synthetic scaffolds, there is no study in the literature that compares the osteoinductive capacity of the two to the best of our knowledge. Our findings revealed that ASP templated peptides conjugated with pNF had more potential in inducing osteogenesis when used in short peptides with the same number of repeats as GLU. There are a number of studies emphasizing the importance of enhancing the bioactivity of synthetic scaffolds for better bone tissue regeneration. It is believed that such findings will help researchers to design better biomimetic scaffolds to be translated into the clinic to improve bone healing processes.

## Electronic supplementary material

Supplementary Figures
