# Supplementary material for: Aspartic and Glutamic Acid Templated Peptides Conjugation on Plasma Modified Nanofibers for Osteogenic Differentiation of Human Mesenchymal Stem Cells: A Comparative Study
Source: Sci Rep. 2018 Dec 4;8:17620. doi: 10.1038/s41598-018-36109-5 (PMC6279782; doi:10.1038/s41598-018-36109-5)
Supplement: Supplementary file 1 — Supplementary Figures [file 41598_2018_36109_MOESM1_ESM.doc]

***Supporting Information For***

**Aspartic and Glutamic Acid Templated Peptides Conjugation on Plasma Modified Nanofibers for Osteogenic Differentiation of Human Mesenchymal Stem Cells: A Comparative Study**

Günnur Onak^1^, Mustafa Şen^1^, Nesrin Horzum^2^, Utku Kürşat Ercan^1^, Ziyşan Buse Yaralı^1^, Bora Garipcan^3^, Ozan Karaman^1,4,*^

1. EEEEEE

1. DDDDDD

1. DDDDDDK

D) EEEEEEK

**Figure S1.** Chemical Structure of peptides, A) EEEEEE, B) DDDDDD, C) EEEEEEK, and D) DDDDDDK.


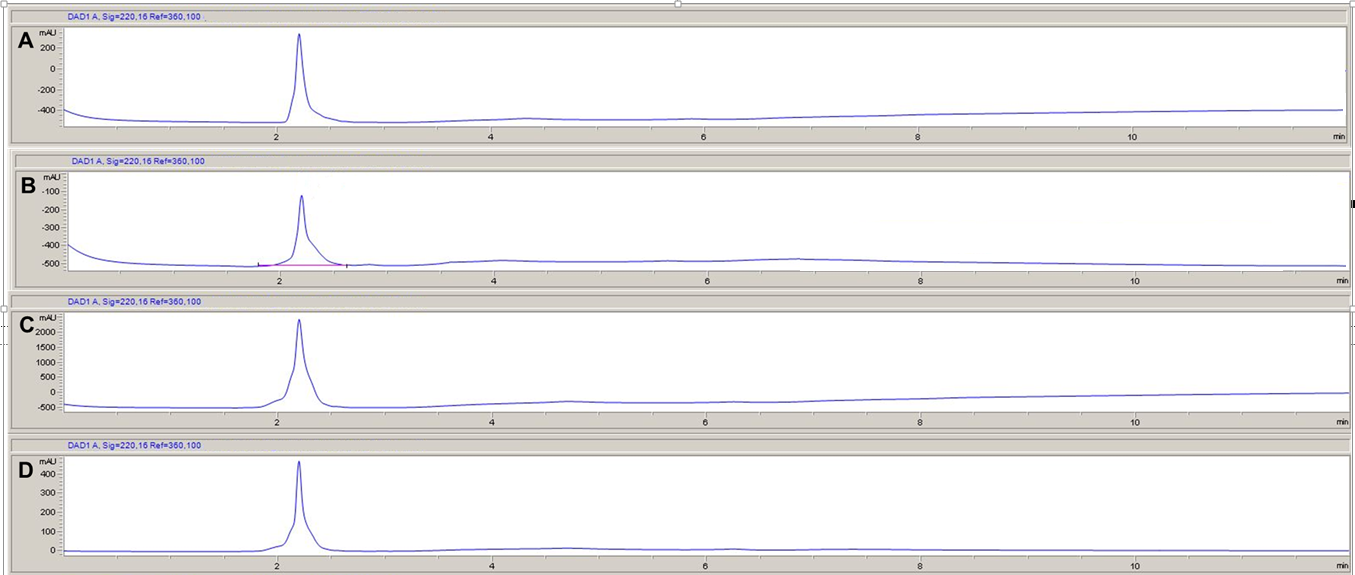


**Figure S2.** Liquid chromatography spectra of A) DDDDDD, B) EEEEEE, C) DDDDDDK and D) EEEEEEK

**
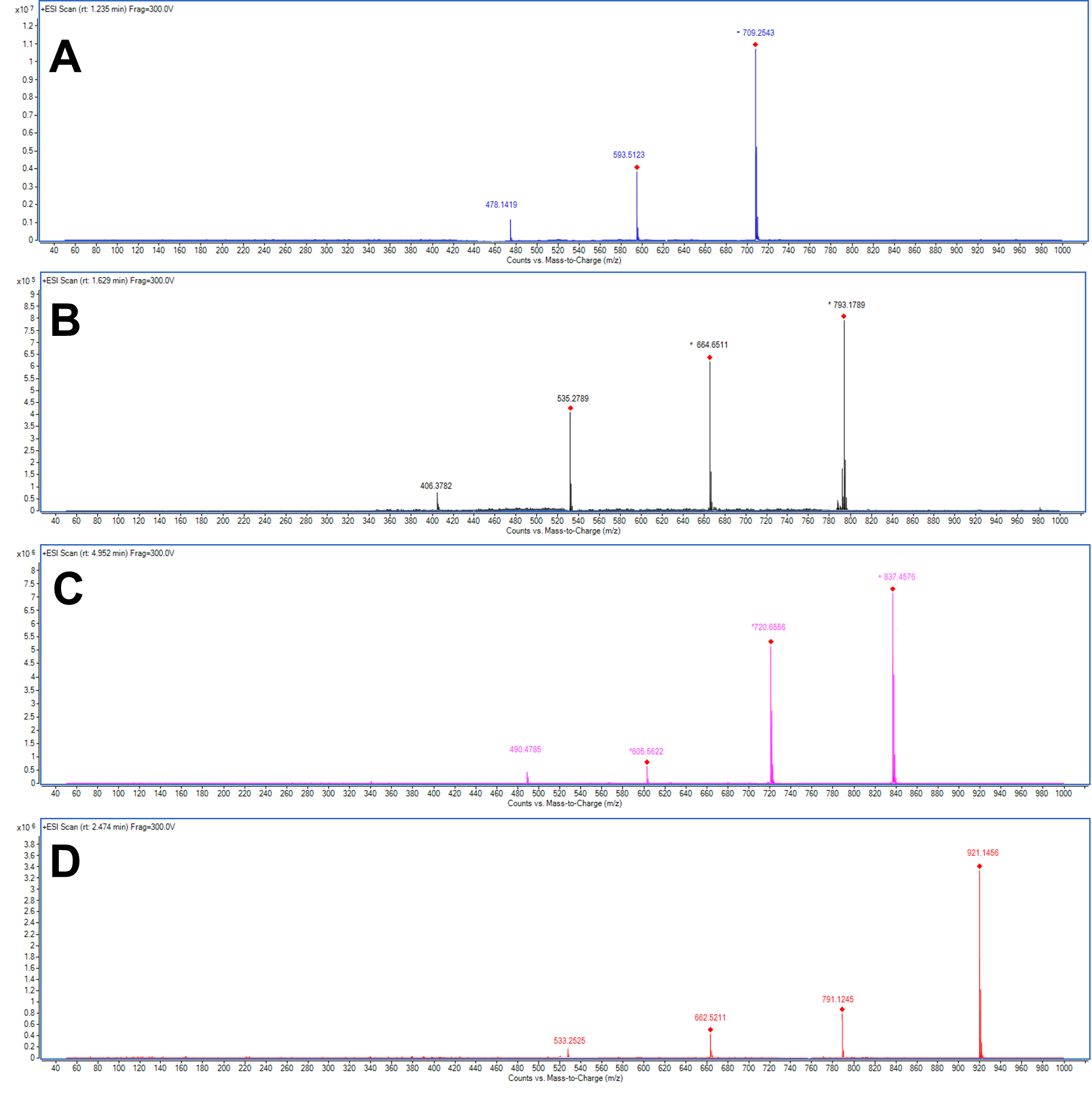
Figure S3.** Ioniztion mass spectra of A) DDDDDD, B) EEEEEE, C) DDDDDDK and D) EEEEEEK

**Supplementary Table S1.** Sequences, observed and calculated molecular weight of synthesized peptides

| Name | Sequence | Calculated | Observed |
| --- | --- | --- | --- |
| ASP | DDDDDD | 708.55 | 709.25 |
| GLU | EEEEEE | 792.71 | 793.17 |
| ASPK | DDDDDDK | 836.72 | 837.45 |
| GLUK | EEEEEEK | 920.88 | 921.14 |

**
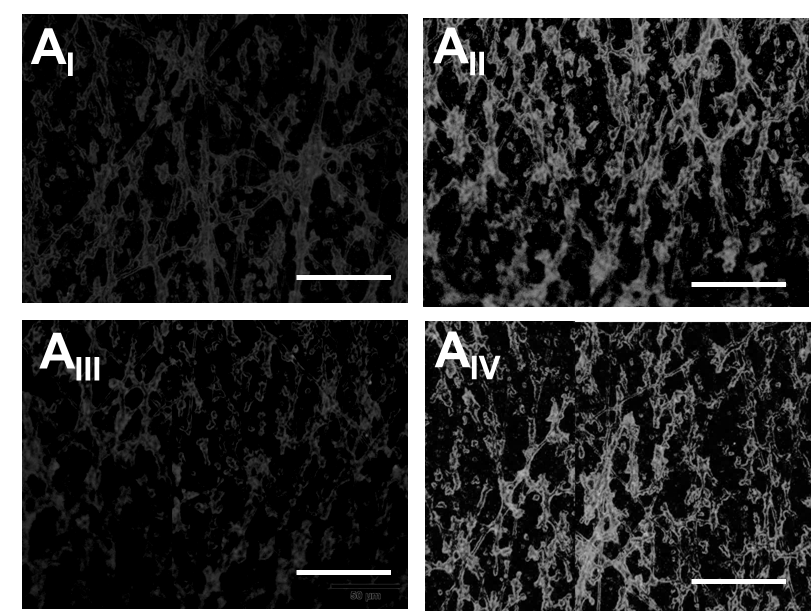
**

**Figure S4.** Grayscale microscopy images of non-CAP treated fluoresceinisothiocyanate (FITC) labeled glutamic acid peptide conjugated NF (GLU-NF) (A_I_ ), CAP treated FITC labeled glutamic acid peptide conjugated NF (GLU-pNF) (A _II_), non-CAP treated fluorescein isothiocyanate (FITC) labeled aspartic acid peptide conjugated NF (ASP-NF) (A_III_),CAP treated FITC labeled aspartic acid peptide conjugated NF (ASP-pNF) (A_IV_) (Scale bar represents 50µm).

**Figure S5.** ATR spectra for glutamic acid (GLU) and aspartic acid (ASP)

**Figure S6.** C1s high-resolution spectra (A_I_, A_II_) of ASP-NF and ASP-pNF (CAP treatment time is 45 seconds).
